# Remifentanil pretreatment ameliorates H/R-induced cardiac microvascular endothelial cell dysfunction by regulating the PI3K/Akt/HIF-1α signaling pathway

**DOI:** 10.1080/21655979.2021.1969843

**Published:** 2021-10-06

**Authors:** Xiaojun Li, Zhenping Gui, Huizi Liu, Shaojie Qian, Yanan Jia, Xiaopan Luo

**Affiliations:** a Department of Anesthesiology, Zhejiang Provincial People’s Hospital, Affiliated People’s Hospital, Hangzhou Medical College, Hangzhou City, Zhejiang Province, P.R. China; b Department of Anesthesiology, Linan Qingshan Lake Hospital of Traditional Chinese Medicine, Hangzhou City, Zhejiang Province, P.R. China

**Keywords:** Remifentanil, myocardial ischemia, hypoxia/reoxygenation, PI3K/Akt/HIF-1α, cardiac microvascular endothelial cells

## Abstract

Restoration of blood supply through medical or surgical intervention is a commonly adopted method for acute myocardial ischemia, but is also a trigger for cardiac ischemia/reperfusion injury. Studies have shown that remifentanil (REM) displays cardioprotective effects. In this study, the effects of REM on HCMEC viability were examined before and after the induction of H/R using Cell Counting Kit-8 assays. Wound healing and Matrigel angiogenesis assays were performed to assess HCMEC migration and angiogenesis, respectively. Commercial kits and western blotting were used to determine the endothelial barrier function of H/R-stimulated HCMECs with or without REM treatment. The expression of PI3K/Akt/hypoxia-inducible factor-1α (HIF-1α) pathway-related proteins was detected by western blotting. After pre-treatment with PI3K/Akt, the effects of REM on H/R-induced HCMEC injury were examined. We found that pre-treatment with REM displayed no impact on HCMEC viability under normal conditions but noticeably improved cell viability following H/R. The migratory abilities and tube-like structure formations of H/R-stimulated HCMECs were both enhanced by REM in a concentration-dependent manner. REM also decreased the permeability of H/R-stimulated HCMECs and upregulated the expression of tight junction proteins. Furthermore REM increased the expression of PI3K/Akt/HIF-1α signaling-related proteins in HCMECs. Inhibition of PI3K/Akt rescued REM-enhanced HCMEC function under H/R condition. Therefore, the present study demonstrated that REM pretreatment ameliorated H/R-induced HCMEC dysfunction by regulating the PI3K/Akt/HIF-1α signaling pathway.

## Introduction

Myocardial ischemia is a pathophysiological condition in which the coronary artery is deficient in blood and oxygen supply to the myocardium due to coronary artery stenosis (primarily caused by atherosclerosis), spasm or embolism [1,2]. In numerous pathological processes leading to heart failure, myocardial ischemia displays the highest prevalence and, thus, represents a serious danger to the health of patients with cardiovascular diseases [3]. As for current therapeutic strategies for myocardial ischemia, treatment in the acute phase promotes the rapid release of the ischemic condition, such as improvement of myocardial blood and oxygen supply using drugs that dilate the coronary artery (e.g., nitroglycerin) or coronary recanalization by minimally invasive or surgical procedures [4]. Although coronary reperfusion therapy effectively reduces myocardial ischemic injury and prevents further tissue damage, cases of reperfusion injury are not clinically rare and occur due to free radical formation, activation of autophagy, calcium overload and neutrophil infiltration into the infarct zone during the process [5–7]. Besides these factors, other physiological processes implicated in the mechanism underlying reperfusion injury are still being explored. Cardiac ischemia/reperfusion (I/R) injury impairs endothelial barrier function, increases endothelial permeability and causes cell swelling, leading to microthrombosis and microvascular obstruction that block the blood supply to the heart [8–10]. Therefore, effective management of I/R injury is important for the treatment of myocardial ischemia.

Remifentanil (REM), a mu-opioid receptor agonist of fentanyl, can quickly reach the blood-brain barrier in the human body and is mostly used as an intra-operative analgesic [11]. Studies suggest that REM may display cardioprotective effects, in that concomitant use of REM in patients undergoing cardiac surgery has been clinically shown to be associated with reduced cardiac troponin release, time of mechanical ventilation and length of hospital stay [12,13]. Moreover, mice preconditioned with REM have been reported to display a much lower risk of myocardial injury following I/R procedures, whereas hyperglycemia-induced oxidative stress could diminish REM cardioprotection by damaging the Caveolin-3-regulated PI3K/Akt and Janus kinase 2 (JAK2)/Stat3 signaling pathways [14]. The present study investigated a similar cardioprotective effect of REM on cardiac microvascular endothelial cells *in vitro*.

Based on the results of the above literature review, we speculate that REM has therapeutic effect on myocardial ischemia-reperfusion injury. Therefore, in this paper, we discussed the effect and mechanism of H/R-induced cardiac microvascular endothelial cell dysfunction. This study provides a solid theoretical basis for REM as a new adjuvant for the treatment of myocardial ischemia.

## Materials and methods

### Cell culture, model induction, and drug treatment

Human cardiac microvascular endothelial cells (HCMECs) were obtained from ScienCell Research Laboratories, Inc. and cultured in Endothelial Cell Medium (ScienCell Research Laboratories, Inc.) with 5 mmol/l glucose (Gibco; Thermo Fisher Scientific, Inc.) and 10% FBS (Gibco; Thermo Fisher Scientific, Inc.) at 37°C with 5% CO_2._ To perform hypoxia/reoxygenation (H/R), HCMECs were incubated in an anaerobic chamber with 95% N_2_ and 5% CO_2_ at 37°C. Prior to hypoxia, the culture medium was replaced with glucose free and serum free Endothelial Cell Medium for 3 h. Then, cells were transferred to FBS‐containing Endothelial Cell Medium and cultured under normal conditions for 2 h. As for REM administration, cells were pretreated with 0.625, 1.25 or 2.5 μm REM for 1 h prior to H/R stimulation. To inhibit PI3K/Akt expression, LY294002 (10 μM) was selected for pretreatment of HCMECs for 1 h prior to H/R.

### Cell counting Kit-8 (CCK-8) assay

Cells (2 x 10^4^ cells/mL) were cultured in 96-well plates at 37°C. After corresponding treatments for different groups, CCK-8 reagent (Beyotime Institute of Biotechnology) was added into each well for 2 h at 37°C. The optical density was measured at a wavelength of 450 nm [15].

### Wound healing assay

Cells were seeded into 12-well plates (2 x 10^5^ cells/well) and treated corresponding to each group. A scratch was made in the confluent cell monolayer using a 10-µl pipette tip. Subsequently, the cells were cultured in serum-free Endothelial Cell Medium. An inverted light microscope (Olympus Corporation; magnification, x100) was used to observe the wounds at 0 and 24 h. The wound healing rate was calculated using ImageJ software (version 1.46; National Institutes of Health). The rate of wound healing = [(the wound width of 0 h – 24 h)/0 h wound width] × 100% [16].

### Matrigel angiogenesis assay

Matrigel (Shanghai Shanran Biotechnology Co., Ltd.) was maintained in a stationary state at 4°C overnight, and became yellow and gelatinous the following day. Subsequently, 70 μl Matrigel (0.5 mmol/l) was added to a pre-cooled 96-well plate. Cell suspensions (1x10^5^ cells/ml) were inoculated in the Matrigel-coated culture well for 18 h. A total of three randomly selected visual fields were visualized in each well. The average length and number of tube-like structures in each visual field were calculated and recorded [17].

### Transendothelial permeability evaluation

The endothelial function of HCMECs was detected using the CultreCoat® 24 Well *In Vitro* Vascular Permeability kit (Trevigen, Inc.) according to the manufacturer’s protocol. Briefly, cells were seeded onto a membrane in complete growth medium to allow proliferation and adhesion prior to treatment. FITC-Dextran was added and allowed to permeate into the plate well through defective cell adhesion. The fluorescence intensity was measured using a microplate reader [18].

### Western blot

Protein samples were prepared using lysis buffer, then loaded and electrophoresed with the running gel. Transfer buffer was used for protein transferring. Following blocking with 5% BSA, the membranes were incubated with the following primary antibodies: Anti-zonula occludens-1 (ZO-1; cat. no. 5406; Cell Signaling Technology, Inc.), anti-vascular endothelial (VE)-cadherin (cat. no. 2158), anti-Claudin-5 (cat. no. sc-374,221; Santa Cruz Biotechnology, Inc.) and GAPDH (cat. no. 5174). Subsequently, the membranes were incubated with a Goat Anti-Rabbit IgG H&L (Alexa Fluor® 488) secondary antibody (cat. no. ab150077; Abcam). Protein blots were visualized using chemiluminescence.

### Statistical analysis

Comparisons among among 3 and more groups were analyzed using one-way ANOVA followed by Tukey’s post hoc test and only two groups were compared with unpaired t test. Statistical analyses were performed using GraphPad Prism software (version 7; GraphPad Software, Inc.). All values are presented as the mean ± standard deviation, and P < 0.05 was considered to indicate a statistically significant difference.

## Results

### REM preconditioning enhances HCMEC viability after H/R

**Figure 1.**
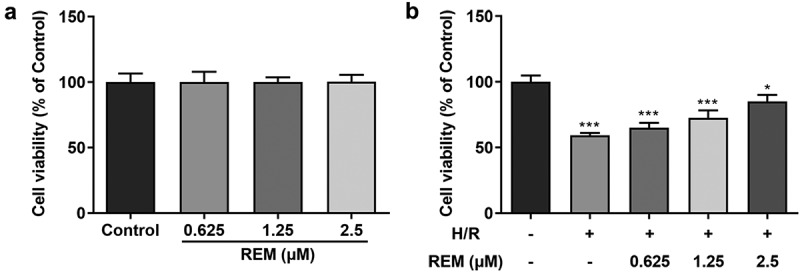
**REM preconditioning enhances the viability of HCMECs after H/R** The viability of HCMECs pre-treated with different concentrations of REM, detected under normal conditions (a) and under H/R conditions (b), detected with CCK-8. *P < 0.05, ***P < 0.001 vs Control

The effect of REM on HCMEC viability in the absence of H/R was examined first. Cells treated with different concentrations of REM exhibited no significant alterations in cell viability (Figure 1a). However, under H/R conditions, HCMECs pretreated with REM exhibited a concentration-dependent increase in viability compared with the model group (Figure 1b).

### REM preconditioning promotes the migration and angiogenesis of HCMECs after H/R

**Figure 2.**
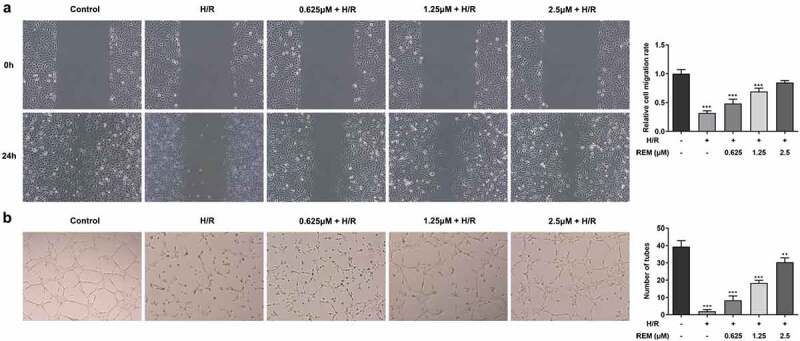
**REM preconditioning promotes the migration and angiogenesis of HCMECs after H/R** The migration (a) and tube-like structure formation (b) abilities of HCMECs pre-treated with different concentrations of REM following H/R, detected by wound healing assay and Matrigel angiogenesis assay. ** P < 0.01, ***P < 0.001 vs Control

The effect of REM on HCMEC migration during H/R was assessed. Cell migration in the H/R group was significantly slower compared with that in the control group, and REM increased cell migration during H/R in a concentration-dependent manner (Figure 2a). Additionally, the Matrigel angiogenesis assay results showed that H/R impaired the tube formation ability of HCMECs in the model group, whereas pretreatment with REM improved HCMEC tube formation in a concentration-dependent manner (Figure 2b).

### REM preconditioning decreases the permeability of HCMECs after H/R and upregulates tight junction protein expression

**Figure 3.**
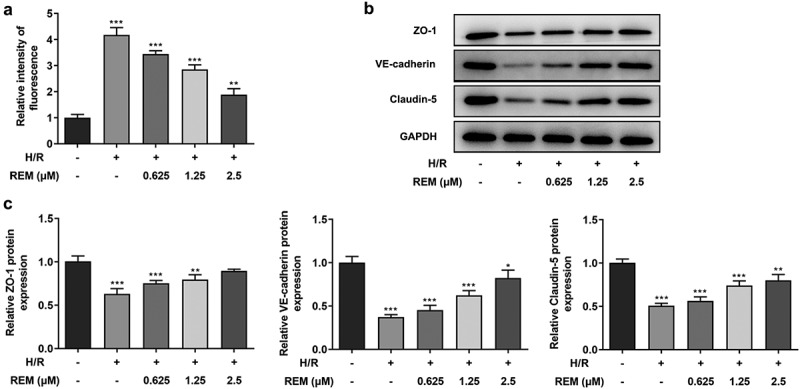
**REM preconditioning decreases the permeability of HCMECs after H/R and upregulates tight junction protein expression** (a) The permeability of HCMECs pre-treated with different concentrations of REM following H/R, detected by in vitro vascular permeability kit. The intensity of fluorescence reflects the degree of cell permeability. (b-c) The expression of tight junction proteins in HCMECs pre-treated with different concentrations of REM following H/R, detected by western blot. *P < 0.05, ** P < 0.01, ***P < 0.001 vs Control

The *in vitro* vascular permeability assay results showed a notable increase in fluorescence intensity in the model group compared with that in the control group, whereas pretreatment with REM gradually reduced the fluorescence intensity with increasing concentrations (Figure 3a). The aforementioned results indicated that REM preconditioning improved the endothelial barrier function of HCMECs under H/R conditions. Furthermore, a decline in the expression levels of tight junction proteins (ZO-1, VE-cadherin and Claudin-5) was detected via western blotting in H/R-stimulated HCMECs, which also demonstrated that protein expression levels in cells pretreated with REM gradually increased with increasing concentrations (Figure 3b and c).

### REM preconditioning regulates the PI3K/Akt/HIF-1α signaling pathway in HCMECs

**Figure 4.**
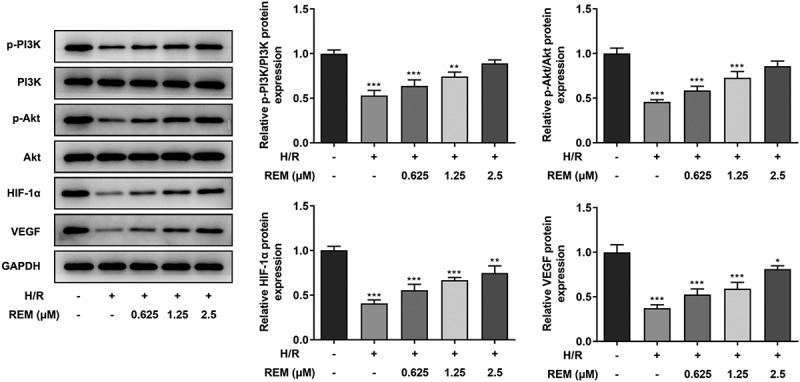
**REM preconditioning regulates the PI3K/Akt/HIF-1α signaling pathway in HCMECs** The expression of relevant proteins to the PI3K/Akt/HIF-1α signaling pathway in HCMECs pre-treated with different concentrations of REM following H/R, detected by western blot. *P < 0.05, ** P < 0.01, ***P < 0.001 vs Control

To identify the association between REM and the PI3K/Akt/HIF-1α signaling pathway, western blotting was performed to detect the expression of relevant proteins [phosphorylated (p)-PI3K/PI3K, p-Akt/Akt, HIF-1α and VEGF] in the control, model and REM treatment groups. Protein expression levels were all much lower in the model group compared with those in the control group, and REM pretreatment elevated the expression levels in HCMECs stimulated with H/R in a concentration-dependent manner (Figure 4).

### PI3K/Akt inhibition rescues REM-induced HCMEC viability, migration and angiogenesis after H/R

**Figure 5.**
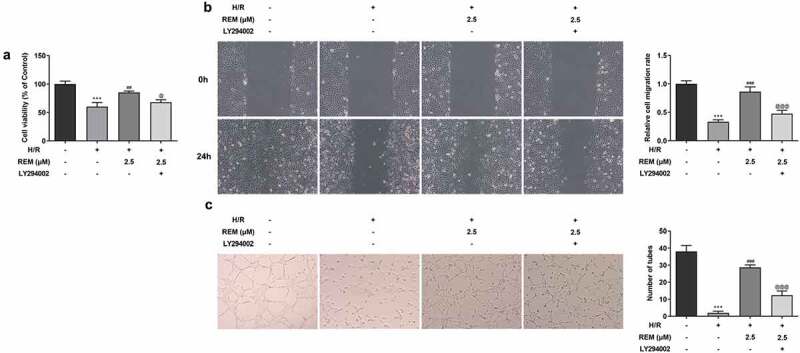
**PI3K/Akt inhibition rescues REM-promoted HCMEC viability, migration and angiogenesis after H/R** The viability (a), migration (b) and tube-like structure formation (c) of HCMECs treated with REM following H/R in the presence of LY294002, correspondingly detected by CCK-8, wound healing and Matrigel angiogenesis assay. ***P < 0.001 vs Control; ##P < 0.01, ### P < 0.001 vs Model; @P < 0.05, @@@P < 0.001 vs H/R+ REM

The aforementioned results demonstrated the protective effect of REM on HCMECs at a concentration of 2.5 μM; therefore, 2.5 μM REM was used for the subsequent experiments. To investigate whether REM benefited HCMEC function during H/R via the PI3K/Akt/HIF-1α signaling pathway, cells were pretreated with the PI3K/Akt inhibitor LY294002 for further experiments. REM-induced increases in HCMEC viability during H/R were decreased in the presence of LY294002 pretreatment (Figure 5a). Pretreatment with LY294002 also rescued REM-induced HCMEC migration during H/R (Figure 5b). Additionally, the REM-induced tube formation abilities of H/R-stimulated HCMECs were significantly impaired in the presence of LY294002 (Figure 5c).

### PI3K/Akt inhibition reverses the protective effect of REM preconditioning on HCMEC endothelial barrier function after H/R

**Figure 6.**
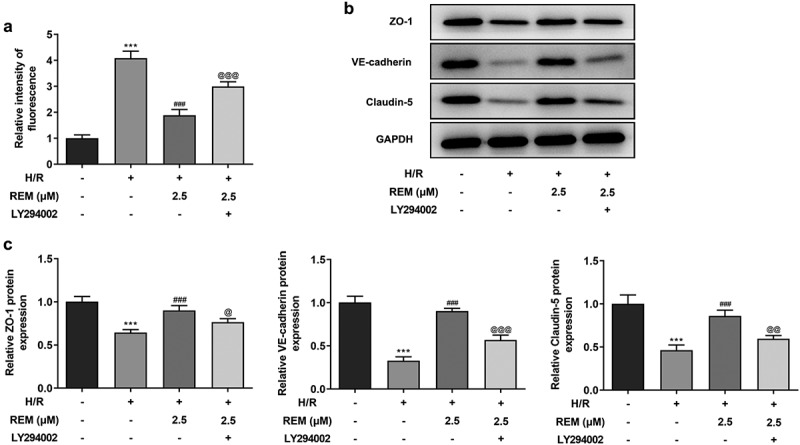
**PI3K/Akt inhibition reverses the protective effect of REM preconditioning on HCMEC endothelial barrier function after H/R** (a) The permeability of HCMECs treated with REM following H/R in the presence of LY294002, detected by in vitro vascular permeability kit. The intensity of fluorescence reflects the degree of cell permeability. (b-c) The expression of tight junction proteins in HCMECs treated with REM following H/R in the presence of LY294002, detected by western blot. ***P < 0.001 vs Control; ### P < 0.001 vs Model; @P < 0.05, @@P < 0.01, @@@P < 0.001 vs H/R+ REM

To further examine the role of PI3K/AKT signaling pathway in the protective effect of REM preconditioning on HCMEC endothelial barrier function, *in vitro* vascular permeability assays showed that H/R-stimulated HCMECs treated with REM displayed upregulated fluorescence intensity in the presence of LY294002 compared with the REM + H/R group (Figure 6a). REM-induced upregulation of the expression of tight junction proteins (ZO-1, VE-cadherin and Claudin-5) was also downregulated by LY294002 pretreatment in HCMECs following H/R (Figure 6b and c).

## Discussion

Myocardial ischemia is a condition in which insufficient blood and oxygen supply to the myocardial tissues results in abnormal energy metabolism of the myocardium and an inability to support the normal functioning of the heart [19]. With improvements in living standards, the prevalence of myocardial ischemia in China has been increasing year by year in the past decades, and it has become a frequently occurring disease in the elderly [20]. There are also clinical cases of young patients between the ages of 20 and 30 showing signs of myocardial ischemia or being diagnosed with coronary heart disease, the most common cause of myocardial ischemia [21,22]. Microvascular obstruction, damaged myocardium, endothelial edema and endothelial sloughing are the frequent outcomes and aggravators of myocardial infarction [23]. Therefore, repairing the function of cardiac microvascular endothelial cells is important for the treatment of myocardial ischemia.

REM is a mu-opioid receptor agonist of fentanyl that is primarily used as an intra-operative analgesic. The analgesic effect of REM is strong enough to be used in a variety of surgeries, including coronary artery bypass surgery [12,24]. Emerging evidence in recent years suggested that REM may display cardioprotective effects in view of reports on how the activation of opioid receptors could potentially protect against myocardial I/R injury in myocardial salvage [25–27]. In addition, a previous study demonstrated that REM-pretreated human cardiomyocytes display an improved resistance to cell senescence and necroptosis induced by hypoxia [28]. In a rat model of isoproterenol-induced myocardial injury, REM effectively improved cardiac dysfunction, lipid peroxidation and immune disorder by blocking the JNK/NF-κB p65 pathway [29]. Moreover, it has been reported that postconditioning with REM could activate autophagic reflux blocked by H/R in H9c2 cardiomyocytes, thus preventing H/R-induced cardiomyocyte injury [30]. The present study first established the safety of using REM in HCMECs and no cytotoxicity was observed. The results also demonstrated that REM preconditioning increased HCMEC viability under H/R conditions.

Microvascular dysfunction in cardiac I/R injury and various other diseases generally presents as inflammation, reduced microvascular flow and impaired angiogenetic and self-repairing abilities [31,32]. Among these, the abnormal microvascular angiogenesis involves insufficient cardiac microvascular endothelial cell migration [33]. In the present study, REM preconditioning promoted the migration of HCMECs and improved their ability to form tube-like structures under H/R conditions. In myocardial I/R injury, insufficient secretion of endothelium-derived relaxing factor and excessive secretion of endothelin-1 in myocardium microvascular endothelial cells leads to increased cell permeability [34,35], further resulting in microvascular endothelial edema, which is part of the pathophysiological transition of reversible ischemic myocardial injury to irreversible injury. ZO-1, VE-cadherin and Claudin-5 are important mediators of endothelial adherence junction and angiogenesis, and are vital in maintaining the blood-brain barrier balance in ischemic stroke [36–38]. The results of the present study showed that REM preconditioning improved the endothelial barrier function by decreasing cell permeability and upregulating the expression of ZO-1, VE-cadherin and Claudin-5. The aforementioned results collectively demonstrate the beneficial effects of REM on the viability, migration, angiogenesis and permeability of HCMECs following H/R.

According to a previous study, REM can repair post-traumatic osteoarthritis cartilage damage by inhibiting PI3K/Akt/NF-κB phosphorylation, reducing cartilage matrix degradation and inhibiting IL-1β-induced apoptosis of articular chondrocytes [39]. Mice preconditioned with REM have been reported to display a much lower risk of myocardial injury following I/R procedures, whereas hyperglycemia-induced oxidative stress could diminish REM cardioprotection by damaging the Caveolin-3-regulated PI3K/Akt and JAK2/Stat3 signaling pathways [14]. Additionally, PI3K/Akt regulated by Ginsenoside Re could serve an alleviative role in high glucose-induced RF/6A retinal endothelial cell injury by inhibiting the HIF-1α/VEGF signaling pathway [40]. More importantly, long non-coding RNA small nucleolar RNA host gene 1-activated HIF-1α/VEGF has been found to increase the expression of VE-cadherin and MMP2, and facilitate the proliferation and angiogenesis of HUVECs following H/R [41]. Therefore, PI3K/Akt/HIF-1α may serve as a signaling pathway in the mechanism underlying REM-mediated cardioprotection against H/R-induced HCMEC injury. After identifying that REM regulated the expression of PI3K/Akt/HIF-1α in HCMECs, the results of the present study further confirmed that inhibiting PI3K/Akt signaling rescued the ameliorative effects of REM on H/R-induced HCMEC injury by decreasing cell viability, migration and angiogenesis, impairing endothelial barrier function and downregulating the expression of tight junction proteins.

In summary, REM pretreatment ameliorated H/R-induced HCMEC dysfunction by regulating the PI3K/Akt/HIF-1α signaling pathway. The present study elucidated the mechanism underlying the cardioprotective effect of REM in H/R-induced HCMEC injury. REM may serve as a novel adjuvant in the treatment of myocardial ischemia and be used for the management of cardiac ischemia/reperfusion injury.

## Research highlights

Remifentanil ameliorated HCMECs dysfunctionRemifentanil targeted the PI3K/Akt/HIF-1α pathwayRemifentanil may become a novel adjuvant in the treatment of myocardial ischemia
